# Clinical observations on infliximab treatment of infantile onset Takayasu arteritis

**DOI:** 10.1186/s12969-022-00708-4

**Published:** 2022-08-04

**Authors:** Min Kang, Jianming Lai, Dan Zhang, Yingjie Xu, Jia Zhu, Ming Li

**Affiliations:** Department of Rheumatology, Capital Institute of Pediatrics, 2 Yabao Road, Chaoyang District, 100020 Beijing, China

**Keywords:** Takayasu arteritis, Infants, Infliximab, Effectiveness, Safety

## Abstract

**Background:**

There is insufficient evidence on the clinical effectiveness and safety of infliximab (IFX) treatment of Takayasu arteritis (TA) in infants.

**Methods:**

We evaluated the therapeutic effectiveness and safety of IFX in a retrospective case series of 10 infantile TA patients. Observations included assessment of clinical symptoms, laboratory testing, and vascular imaging.

**Results:**

Fever was the presenting symptom for 8 of 10 infants with TA. During acute episodes, leucocyte and inflammatory indices were significantly increased. Vascular imaging showed the most commonly involved arteries to be carotid arteries, abdominal aortas, and coronary arteries (9 cases, 90%). Two weeks after initiating IFX treatment, leukocyte and platelet counts decreased and hemoglobin levels increased. There were statistically significant clinical improvements 6 weeks after starting treatment compared with before treatment (*p* < 0.05). Inflammatory indices decreased 2 weeks after starting IFX treatment compared with before treatment (*p* < 0.05). Vascular lesions began to recover within 1.5-3 months of initiating IFX therapy, and involved vessels significantly recovered within 13 months. Some arteries remained stenotic, with intimal thickening and uneven lumen wall thicknesses. The only adverse event was a treatment-responsive allergic reaction during IFX infusion in one infant.

**Conclusions:**

Fever was the main manifestation of illness and was often accompanied by significantly increased inflammatory indices. IFX treatment was apparently effective and reduced or eliminated need for glucocorticoids. IFX had a reasonably good safety profile.

## Background

Takayasu’s arteritis (TA) is a systemic vasculitis involving the aorta and its main branches that causes chronic arterial inflammation, leading to stenosis or even occlusion of affected arteries. It occurs most frequently in women and is rare in infancy and childhood. However, TA does occur in infancy, generally with multi-vessel involvement, severe systemic inflammation, and a bad prognosis. Glucocorticoids and immunosuppressive agents are currently the drugs of choice for treatment of TA, but their side effects are undesirable and may be unacceptable in infants. Recent clinical trials have demonstrated the efficacy of infliximab (IFX) in both adult and childhood TA [1–5]. However, there are few case reports assessing effectiveness of IFX in infantile onset TA. We report 10 cases of infantile onset TA that were treated with IFX during and after 2016; we describe effectiveness and safety of IFX in a case series.

## Methods

### Patients

To be included in the study, patients must meet diagnostic criteria of childhood TA [6] and have onset of illness during their first year of life. Diagnostic criteria of TA are conventional, CT, or MR angiographic abnormalities of the aorta or its main branches (mandatory criterion), plus at least one of the following five features: (1) decreased peripheral artery pulse and/or claudication of extremities, (2) blood pressure difference > 10 mmHg, (3) bruits over the aorta and/or its major branches, (4) hypertension (relative to childhood normative data), or (5) erythrocyte sedimentation rate > 20 mm per hour or any CRP value above normal.

Patients were excluded from consideration of IFX treatment if they had evidence of acute or chronic active viral, mycoplasma, or fungal infections. Patients were tested for cytomegalovirus nucleic acid, EB virus nucleic acid, Legionella pneumophila serum type I IgM antibody, Chlamydia pneumoniae IgM antibody, adenovirus IgM antibody, respiratory syncytial virus IgM antibody, influenza A and B virus antibody, and parainfluenza virus IgM antibody, and with bacterial blood cultures. Patients with tuberculosis exposure history or who tested positive in the interferon release test for tuberculosis were excluded. Patients testing positive for hepatitis B surface antigen were excluded. We tested immune function and conducted whole exon genome sequencing to exclude patients with congenital immune deficiencies.

Between July 2016 and August 2020, ten infants were diagnosed with TA in our hospital, admitted for treatment, and met our inclusion criteria and had no exclusion criteria. These ten infants formed the case series study group.

### Ethical review

The study was approved by the Ethical Committee of Children’s Hospital Capital Institute of Pediatrics; guardians provided written, informed consent for their infants.

### Treatment

Infliximab, 5-6 mg/kg, was given intravenously at 0, 2, 6, 14, 22, and 30 weeks and, depending on the apparent effect of the treatment, additional IFX injections were given at 8-week intervals. Oral antihistamines were given prior to IFX infusion to prevent acute infusion reactions. Five patients were treated with IFX alone; glucocorticoid (GC) and immunosuppressive therapy was used before IFX was used in another 5 patients, and for these patients, GC doses were adjusted during IFX treatment in accordance with clinical condition.

### Clinical indicators

We abstracted infants’ medical records for age, gender, medical history, physical examination, laboratory tests, cardiac ultrasound, vascular ultrasound, chest and abdomen enhanced CT, and CT angiography (CTA). We recorded time-varying clinical data of temperature, blood pressure, routine blood tests, inflammatory indices, cytokines, and immune functioning. Vascular ultrasound and/or CTA was used to evaluate progression of vascular lesions. We recorded GC and immunosuppressive agent doses administered before and after IFX was started. During IFX treatment, no patients received bacille Calmette-Guéri or other live vaccine.

### Safety evaluation and follow-up

The infants were followed up every 1 to 3 months for 8 to 64 months to record symptoms, liver and kidney functioning, and signs of infection. We conducted follow-up assessments in the hospital, during outpatient visits, and by telephone. We obtained data on height, weight, clinical symptoms, and treatment. No cases were lost to follow-up.

### Statistical analysis

Continuous data are presented as means with standard deviations or ranges. Means were compared by independent-sample *t* tests. A *p* value < 0.05 was considered statistically significant.

## Results

### Baseline characteristics

Ten infants qualified for the study - two boys and eight girls. The ages of onset of TA ranged from 1 month and 17 days to 5 months and 7 days. At the time of diagnosis, the durations of illness were less than 1 month in seven cases and 1–3 months in the other three cases. Before their diagnosis of TA, the patients’ initial diagnoses were fever of unknown origin (*n* = 4), Kawasaki disease (*n* = 3), pneumonia (*n* = 2), and inflammatory bowel disease (*n* = 1). All patients had been initially treated with antibiotics; eight had been treated with intravenous immunoglobulin.

### Clinical manifestation and medication before IFX

The most common clinical manifestation was fever, which was seen in nine cases (90%). Fever was the presenting symptom in eight cases; one other case had a transient fever during the illness onset. Six (60%) infants had hypertension; three (30%) had weak or no detectable pulse; two (20%) had vomiting, and one had transient low-grade fever twice during the illness; no infants had a rash or vascular bruits (Table 1).

**Table 1:** Clinical characteristics and treatment of 10 infants with Takayasu arteritis

Case	Sex	Age at onset (m)	Age at diagnosis (m)	Age treated with IFX (m)	Fever	Hypertension	Vomiting	Rash	Pulselessness	Vascular bruit	Angiographic type	Treatment
1	F	2.5	4	4.3	+	+	+	−	+	−	V	IFX
2	F	2	2.5	2.7	+	−	−	−	−	−	I	IFX
3	F	2.7	3.2	3.5	+	+	−	−	−	−	V	IFX
4	F	1.5	2	2.3	+	+	−	−	−	−	III	IFX
5	M	2	2.7	3	+	+	−	−	+	−	V	IFX
6	F	3	5.2	5.7	+	+	−	−	−	−	V	GC + IFX
7	F	3	3.7	4.2	+	−	−	−	−	−	V	GC + IFX
8	F	5.2	5.7	6.3	+	+	−	−	−	−	V	GC + IFX
9	F	2.2	3	9	+	−	−	−	−	−	V	GC + MTX, IFX
10	M	3	4	7	−	−	+	−	+	−	V	GC + TCZ, GC + IFX

+ represents positive, − represents negative, m means month, *IFX *infliximab, *GC *glucocorticoids, *MTX  *methotrexate, *TCZ *tocilizumab

The most commonly involved vessels were carotid arteries, abdominal aortas, and coronary arteries (9 cases, 90%); thoracic aortas and subclavian aortas (8 cases, 80%); renal arteries (7 cases, 70%); axillary arteries, pulmonary arteries, and descending aortas (6 cases, 60%); and superior mesenteric arteries (4 cases, 40%) (Table 2).

**Table 2:** Changes in laboratory values χ^2^ ± s (P25,P75)

**Treatment**	**Period**	**Laboratory tests**				
		**WBC** (⋅10^9^/L)	**HGB** **(**g/L)	**PLT** **(**⋅10^9^/L)	**CRP** (mg/L)	**ESR** **(**mm/1 h)
IFX	Before	19.31 ± 3.22 (17.56,22.6)	95.8 ± 11.26 (92,99)	828.6 ± 192.94 (692,870)	66.8 ± 39.32 (41,106)	79 ± 40.51 (58,119)
	After 2 weeks	14.53 ± 1.82 (13.38,16,59)	111.8 ± 7.53* (107,120)	441.6 ± 151.15 (384,641)	3.66 ± 2.36 (2.3,5)	17.6 ± 13.15 (7,23)
	*t* value	2.89	2.64	3.53	3.58	3.22
	*p*	0.02	0.03	0.01	0.01	0.01
	the last time follow-up	9.76 ± 2.46 (9.09,10.89)	123 ± 8.66 (120,124)	366.6 ± 104.11 (304,359)	3.66 ± 2.36 (0.78,1.9)	5.2 ± 2.86 (3,7)
IFX + GC	Before	16.07 ± 5.52 (14.38,17.9)	93.4 ± 6.62 (92,98)	664.4 ± 193.16 (516,855)	101.96 ± 33.71 (85,118)	67.8 ± 40.88 (34,80)
	After 2 weeks	10.77 ± 4.49 (8.63,12.33)	119.2 ± 13.95* (108,127)	386 ± 91.59 (359,472)	14.88 ± 11.33 (12,17)	21.2 ± 13.97 (16,29)
	*t* value	1.67	3.74	7.5	5.48	2.41
	*p*	0.13	0.01	0.01	0.01	0.04
	the last time follow-up	9.38 ± 1.71 (8.43,10.75)	123.2 ± 6.97 (118,128)	329.4 ± 59.23 (298,348)	2.52 ± 1.00 (2,3.4)	5.2 ± 3.37 (3,6)

*-after 6 weeks, *WBC *leukocyte, *HGB *hemoglobin, *PLT *platelet, *CRP *C-reactive protein, *ESR *erythrocyte sedimentation rate, *IFX *infliximab, *GC *glucocorticoids

**Fig. 1:**
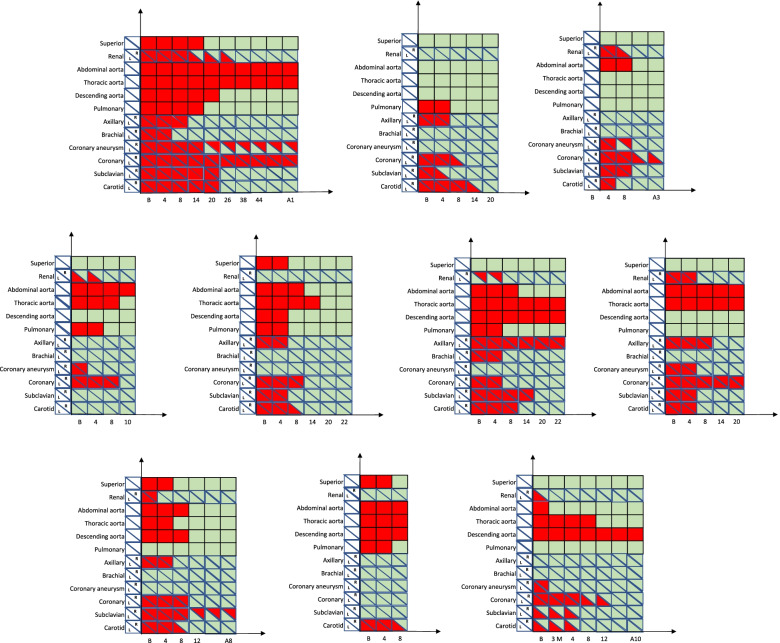
Involved arteries before and after treatment, in 10 infants with Takayasu arteritis. Legend: Red means involved, Green means not involved or recovered. Note: L = Left, R = Right, B: Means before use of medicines, A1: Means 20 months after stopping IFX, A3: Means 25 months after stopping IFX, A8: Means 14 months after stopping IFX, A10: Means 4 months after stopping IFX, 3 M Means 3 months of GC+TCZ, (IFX=infliximab, GC=glucocorticoids, TCZ=tocilizumab)

Five of the infants were treated with IFX alone. The other five infants had been treated with GC (1-1.5 mg/kg/day) prior to receiving IFX, which was started because their inflammation indexes did not decrease and/or vascular imaging showed extensive involvement or no improvement. Cases 6, 7, and 8 had been treated with GC (1 mg/kg/day) prior to receiving IFX; their inflammation indexes decreased slightly. After combination therapy with IFX, GC doses were decreased - quickly at first, then gradually, discontinuing by 12 weeks into therapy in 2 cases and 16 weeks in the other case. The Case 9 infant had been treated with prednisone (1.5 mg/kg/day). Temperature and inflammatory indexes quickly returned to normal. After oral administration of prednisone for 4 months, the dose was reduced to 5 mg/day for 3 months and was combined with methotrexate (MTX) for 4 more months. However, CTA showed that the celiac trunk artery and the left iliac artery were thinner than before, suggesting that the vascular lesion was worsening. The infant had slow growth and development. IFX therapy was added and prednisone was reduced and then discontinued in 2 weeks. For the Case 10 infant, oral GC (1 mg/kg/day) was used in combination with anti-interleukin 6 receptor antibody (tocilizumab) (12 mg/kg, every two weeks) for 3 months. The coronary arteries showed significant improvement, but CTA showed no more improvement than was seen at the beginning of the disease; therapy was changed to IFX(Fig. 1). No abnormalities were found in ophthalmic examinations of the patients.

#### Clinical manifestations and outcomes after IFX treatment

In the five patients treated with IFX alone, fever was controlled in 1–2 days. Blood pressures decreased to normal ranges after 3–8 months of IFX without use of antihypertensives. Three infants continued to have weak pulses that did not improve with IFX.

Coronary arteries were reexamined one and a half months after initiation of IFX; coronary artery examinations and vascular imaging were performed at 4, 8, 14, 20, 26, and 32 months. After one and a half months of treatment, coronary ultrasound showed that the diameters of affected coronary arteries decreased. After four months, vascular ultrasound showed that the average thickness of affected arterial walls decreased, the number of diseased vessels decreased, and vessel wall thickening decreased. After 8 months, the affected vessels improved to a great extent and in some cases returned to normal. With continued treatment, vascular wall lesions ultimately improved, but some complications remained. Affected artery lumens were uneven; there was some vascular dilatation and stenosis, especially of the thoracic and abdominal aorta. Subjects did not worsen clinically, and vascular narrowing showed some improvement (Table 2). None of the patients had heart failure or aortic regurgitation.

The growth and development of nine infants with TA was similar to age-matched healthy infants after 8–64 months of follow-up. In one infant treated with GC combined with MTX, the height was less than the 3^rd^ percentile of same-age healthy infants. At eight months, height was at the 5^th^ percentile.

#### Laboratory tests

At onset of TA, leukocyte and platelet counts were high, hemoglobin was low, and inflammatory indexes were high, suggesting that all ten cases were in the active, acute stage of TA. Leukocytes and platelet counts decreased, and hemoglobin increased after two weeks of IFX treatment. By six weeks, these laboratory values had improved compared with values before treatment (*p* < 0.05). Two weeks after IFX treatment started, the inflammatory indexes of C-reactive protein and erythrocyte sedimentation rate decreased significantly compared with before treatment (*p* < 0.05) (Table 2).

### Safety evaluation and follow-up

One patient, Case10, had a red rash on her face and chest during the 4^th^ and 7^th^ infusion of IFX. The rash subsided when the infusion was stopped, and no adverse events occurred when the infusion was resumed. During the first 15 min of her 8^th^ infusion, she had vomiting and was pale, although with normal blood pressure. Vomiting stopped when the infusion was stopped. In addition, she had a red rash that subsided 30 min after oral antiallergic drugs were given. We considered this an allergic reaction, and the infusion was not restarted. No other infants had adverse reactions. No cases of severe infection or pneumonia were seen during treatment or follow-up. In Case 1, IFX was used for 44 months (24 infusions total) and then MTX was added. In cases 2 and 7, IFX was used for 20 months (12 infusions for each infant), and MTX was added in Case 7 during IFX treatment. In Cases 3, IFX was used for 8 months (6 infusions). In Case 4, IFX was used for 10 months (7 infusions). In Case 5 and 6, IFX was used for 22 months (13 infusions for each infant), and MTX was added in Case 6. In cases 8 and 10, IFX was used for 12 months (8 infusions for each infant), and MTX was added in Case 10. In Case 9, IFX was used for 8 months (6 infusions). One to three months after starting IFX treatment, inflammatory indexes and hematological tests of all cases returned to normal. Routine hematologic tests and inflammatory indexes remained normal during follow-up.

## Discussion

Takayasu arteritis usually occurs among females 40 years of age and younger, including children, but is rare during infancy. Because clinical manifestations are not specific, TA is difficult to diagnose. We studied ten infants diagnosed with TA, most of whom were initially diagnosed with an infection or incomplete Kawasaki disease and who had been treated with antibiotics or intravenous immunoglobulin. However, with uncontrollable fever and inflammation and observation of coronary artery involvement, TA was considered as a diagnosis in these cases and was confirmed with further laboratory testing and vascular imaging. There are few reports of treatment results in the scientific literature, especially treatment of infantile TA, which is rare, making our treatment results useful for clinicians and researchers.

TA is usually chronic with a linear course or a remitting-relapsing course. The inflammatory process causes thickening of arteries with narrowing or occlusion of the lumen. Glucocorticoids are the mainstay of treatment. Because glucocorticoids alone do not always achieve and maintain remission, approximately half of TA patients require immunosuppressive agents [4, 6–9]. In contrast with childhood TA, vascular involvement is highly variable in infants and carries a worse prognosis, requiring high-dose, long-duration GC use. Unfortunately, glucocorticoids significantly affect growth and development of infants – a side effect that cannot be ignored. For example, in our study, one infant had been treated with glucocorticoids for 7 months, affecting growth and development and necessitating consideration of alternative therapy.

Clinical manifestations of TA are nonspecific in infants and children. Fever and inflammation are the most common manifestations, and the abdominal aorta is the most commonly involved vessel [5, 10–15]. In our series, eight (80%) cases had fever as the first symptom and most prominent clinical manifestation. Laboratory analyses showed increased leukocytes and inflammatory indexes. Vascular imaging showed that large and medium arteries were involved. The most commonly involved arteries were carotid arteries, abdominal aortas and coronary arteries (9 cases, 90%). Thoracic aortas and subclavian aortas (8 cases, 80%); renal arteries (7 cases, 70%); axillary arteries, pulmonary arteries, and descending aortas (6 cases, 60%); and superior mesenteric arteries (4 cases, 40%) were also commonly involved. All 10 infants were in active disease states at the time of diagnosis with widespread vascular involvement, suggesting that the infants’ conditions were serious and had poor prognoses.

Tumor necrosis factor alpha (TNF-α) is implicated in TA inflammation. IFX is a TNF-α inhibitor that has direct cytotoxicity and induces apoptosis of immune cells. IFX is reported to have significant effectiveness in refractory TA that is resistant to glucocorticoids and immunosuppressants [8, 9, 16–19]. Our observations showed that IFX is also effective for treatment of TA in infants. Five of our cases were treated with IFX alone and five were treated with IFX combined with GC. We found that temperatures rapidly normalized, inflammatory indexes decreased, and vascular lesions gradually improved with IFX therapy. Additionally, IFX enabled a reduction in dose or discontinuation of GC. In cases 1–8, vascular lesions treated with IFX began to resolve within 1.5-3 months. Most of the affected arteries recovered within 13 months, but some remained stenotic, with intimal thickening and uneven lumens that which did not resolve. In Case 9, some vascular lesions worsened during GC and MTX treatment, but the vascular lesions gradually recovered after initiating IFX. In Case 10, the coronary arteries and abdominal aorta improved, but other vascular lesions did not improve during three months of combination GC and tocilizumab therapy. Some vascular lesions gradually recovered after initiating IFX. In 6 infants with hypertension, blood pressure returned to normal after 3–8 months of IFX treatment, showing that treatment with IFX could lead to an inactive disease status. IFX was effective, beneficial, and well-tolerated in all 10 TA infants. In Case 9, growth and development lagged behind normal growth and development, but after stopping glucocorticoids and starting IFX, growth and development improved. Most infants in our study were diagnosed by 3 months of age. Clinical features and vascular lesions improved to varying degrees after IFX treatment. The positive outcomes may be related to early diagnosis and treatment. The most common initial symptom of infantile TA was fever, which usually serves as a prompt to parents to seek timely evaluation of their febrile infant, thus leading to a young age at diagnosis.

Our study showed that IFX has a good safety profile for use in infants. During IFX treatment, only 1 infant had an allergic reaction, which was during the eighth infusion; other infusions were not associated with adverse events. No severe infections were diagnosed during treatment, consistent with previous reports [10, 13, 19, 20]. Early application of IFX in cases 1-8 had no impact on growth and development

Our study had limitations. Our case series had only 10 infants, and the follow-up time was relatively short. Therefore, studies of long-term effectiveness and safety of IFX for infantile TA need to include more infants and have longer follow-up times.

## Conclusions

In infant-onset TA, use of IFX can significantly and rapidly decrease inflammation and resolve clinical features of active disease, improve vascular lesions, and sustain remission for longer time than treatment with glucocorticoids and methotrexate. IFX enables dose reduction or discontinuation of GC, which promotes normal infant growth and development. IFX appears to cure some cases of infantile TA and induces a high rate of clinical remission.

## Data Availability

Available upon request.

## Abbreviations

TA: Takayasu arteritis
IFX: infliximab
GC: glucocorticoid
MTX: methotrexate
CTA: CT angiography
TCZ: tocilizumab
WBC: leukocyte
HGB: hemoglobin
PLT: platelet
CRP: C-reactive protein
ESR: erythrocyte sedimentation rate
